# Identification of a novel lipin homologue from the parasitic protozoan *Trypanosoma brucei*

**DOI:** 10.1186/1471-2180-13-101

**Published:** 2013-05-08

**Authors:** Michel Pelletier, Alyssa S Frainier, Dominic N Munini, Jenna M Wiemer, Amber R Karpie, Jeff J Sattora

**Affiliations:** 1 Department of Biology, The College at Brockport, State University of New York, Brockport, NY, USA

**Keywords:** Kinetoplastid, Lipin, Arginine methylation, Phosphatidic acid phosphatase

## Abstract

**Background:**

Arginine methylation is a post-translational modification that expands the functional diversity of proteins. Kinetoplastid parasites contain a relatively large group of protein arginine methyltransferases (PRMTs) compared to other single celled eukaryotes. Several *T. brucei* proteins have been shown to serve as TbPRMT substrates *in vitro*, and a great number of proteins likely to undergo methylation are predicted by the *T. brucei* genome. This indicates that a large number of proteins whose functions are modulated by arginine methylation await discovery in trypanosomes. Here, we employed a yeast two-hybrid screen using as bait the major *T. brucei* type I PRMT, TbPRMT1, to identify potential substrates of this enzyme.

**Results:**

We identified a protein containing N-LIP and C-LIP domains that we term TbLpn. These domains are usually present in a family of proteins known as lipins, and involved in phospholipid biosynthesis and gene regulation. Far western and co-immunoprecipitation assays confirmed the TbPRMT1-TbLpn interaction. We also demonstrated that TbLpn is localized mainly to the cytosol, and is methylated i*n vivo.* In addition, we showed that, similar to mammalian and yeast proteins with N-LIP and C-LIP domains, recombinant TbLpn exhibits phosphatidic acid phosphatase activity, and that two conserved aspartic acid residues present in the C-LIP domain are critical for its enzymatic activity.

**Conclusions:**

This study reports the characterization of a novel trypanosome protein and provides insight into its enzymatic activity and function in phospholipid biosynthesis. It also indicates that TbLpn functions may be modulated by arginine methylation.

## Background

Arginine methylation is a post-translational modification whose importance and widespread impact has recently begun to be fully appreciated [1-4]. In yeast and mammals, arginine methylation has been associated with a diversity of cellular processes including signal transduction [5,6], RNA transport [7,8] and processing [9-12], protein localization [13-15], and transcription [16]. The effects of arginine methylation on these processes are exerted primarily through the modulation of protein-protein and, less often, protein-nucleic acid interactions [17-20]. Common sites of arginine methylation within proteins include RGG, RG, or RXR motifs [21-23], although methylation of arginine also occurs within other sequence contexts [24]. Catalysis of arginine methylation is carried out by a family of enzymes termed protein arginine methyltransferases [PRMTs). While these enzymes are apparently absent from prokaryotes, putative PRMTs have been identified in the genomes of all eukaryotes examined with the exception of *Giardia lamblia*[1,25,26].

PRMTs are classified into four types. Both type I and II PRMTs catalyze the formation of ω-N^G^ monomethylarginine (MMA). Type I enzymes subsequently synthesize ω-N^G^,N^G^ asymmetrical dimethylarginine (ADMA), while the type II enzymes form ω-N^G^,N’^G^ symmetrical dimethylarginine (SDMA). Type III and type IV enzymes catalyze the formation of only ω-N^G^ monomethylarginine (MMA) or δ-N^G^ monomethylarginine, respectively. In humans, nine PRMTs have been confirmed, most of them being type I enzymes [3]. In contrast to what has been described in humans, only three PRMTs have been described in *Saccharomyces cerevisiae,* one each of type I type II, and the apparently fungal-specific type IV [1]. Most protozoa with the exception of *Giardia* who lacks putative PTMTS, are predicted to possess at least one type I and one type II PRMTs [26].

*Trypanosoma brucei* is a parasitic protozoan and the causative agent of African sleeping sickness in humans and nagana in African livestock. The genome of *T. brucei* predicts the presence of five PRMTs [26], a relatively large number for a single celled organism [1]. These PRMTS, with the exception of the putative type I TbPRMT3, have previously been characterized. TbPRMT1 is the major type I PRMT in *T. brucei*, analogous to its role in yeast and mammals [27]. TbPRMT5 is a type II enzyme homologous to human PRMT5 [28]. TbPRMT7 is a novel, kinetoplastid-specific type III PRMT [29]. Finally, the recently characterized TbPRMT6 is a type I PRMT capable of automethylation [30]. To date, only a few arginine methylproteins have been reported in *T. brucei*. These include the mitochondrial RNA binding proteins RBP16, TbRGG1, TbRGG2, and MRP2. The effects of RBP16 methylation have been characterized. RBP16 is a TbPRMT1 substrate, as shown by *in vitro* methylation assays and the hypomethylated state of RBP16 in TbPRMT1 knockdown cells [31]. Arginine methylation affects the ability of RBP16 to stabilize specific mitochondrial RNAs and exerts both positive and negative impacts on the interaction of RBP16 with different classes of RNAs and ribonucleoprotein complexes [18,31]. In addition, a large number of proteins harboring arginine/glycine rich regions likely to undergo methylation are predicted by the *T. brucei* genome, and several *T. brucei* RNA binding proteins serve as TbPRMT substrates *in vitro*[26-29,32]. This indicates that a large number of proteins whose functions are modulated by arginine methylation await discovery in trypanosomes.

To gain insight into functions of arginine methylation in trypanosome gene regulation, we set out to identify substrates of the major *T. brucei* type I PRMT, TbPRMT1. We performed a yeast two-hybrid screen using the entire TbPRMT1 open reading frame as bait, exploiting the propensity of PRMTs to associate in a relatively stable manner with their substrates [33]. Using this approach, we identified a protein containing two conserved domains found in a family of proteins known as lipins. Lipins are involved in adipocyte development and phospholipid biosynthesis in mammalian and yeast cells. We termed this protein TbLpn. While these two domains, known as N-LIP and C-LIP domains, are found in several mammalian and yeast lipin proteins [34,35], TbLpn possesses no homology to known proteins outside these two domains and is, thus, a kinetoplastid-specific protein. Consistent with the yeast-two-hybrid data, we show that TbLpn interacts *in vivo* with TbPRMT1, and that it is methylated on arginine residues *in vivo*. We also show that, as predicted by the presence of conserved domains, TbLpn displays phosphatidic acid phosphatase activity *in vitro*, and that the two conserved aspartic acid residues present in the C-LIP domain, are essential for enzymatic activity.

## Results

### Identification of TbLpn as a TbPRMT1-interacting protein

To begin to understand the functions of protein arginine methylation in trypanosomes, we sought to identify proteins that interact with the major type I PRMT in *T. brucei*, TbPRMT1. PRMTs tend to associate in a relatively stable manner with their substrates, and several mammalian methylproteins have been identified through protein-protein interaction screens with PRMTs [36,37]. To identify TbPRMT1-interacting proteins, we screened a yeast-two-hybrid library comprised of mixed procyclic (PF) and bloodstream form (BF) *T. brucei* cDNA [38] using the entire TbPRMT1 ORF as bait. Approximately 800 colonies that grew under moderate selection on SD medium (-Trp, -Leu, -His) were selected for more stringent screening on SD medium (-Trp, -Leu, -His, -Ade).

**Figure 1:**
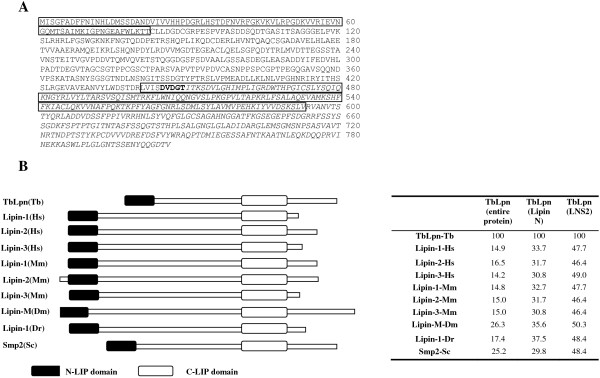
**TbLpn sequence analysis. A**) Shown is the predicted amino acid sequence of TbLpn. The region shown to interact with TbPRMT1 by yeast-two hybrid analysis is shown in italics. The N-LIP and C-LIP domains are boxed. The conserved DxDxT domain is shown in bold. **B**) A schematic representation of TbLpn amino acid sequence aligned with members of the lipin family is shown in the left panel. The degree of amino acid sequence identity between TbLpn and members of the lipin family is shown on the right panel. TbLpn [*T. brucei*, (Tb), accession number AAX78871], Lipin-1 [Human, (Hs), AAH30537], Lipin-2 [Human, (Hs), AAI52449], Lipin-3 [Human (Hs), CAI42978], Lipin-1 [Mouse, (Mm), NP_766538], Lipin-2 [Mouse (Mm), AAH39698], Lipin-3 [Mouse (Mm), EDL06298], Lipin-M [*Drosophila melanogaster*, (Dm), NP_001188884], Lipin-1 [*Danio rerio*, (Dr), AAX19945], Smp2 [*Saccharomyces cerevisiae*, (Sc), BAA00880].

One of the colonies isolated from this screen contained a 1,071-nucleotide insert, which we identified as a fragment of *T. brucei* gene Tb927.7.5450 (http://www.genedb.org) (Figure 1A). The predicted protein encoded by this gene contains an N-LIP domain at its amino terminus, as well as a C-LIP domain extending from amino acid 441–593. These 2 domains are found in a family of proteins known as lipins (Figure 1B). Lipin-1, the first member of this family, was identified in the mouse by positional cloning of the mutant gene responsible for fatty liver dystrophy (*fld*) [39]. In addition, the *fld* mice also exhibit hypertriglyceridemia, increased susceptibility to atherosclerosis, insulin resistance, and peripheral neuropathy [39-41]. Lipin proteins are present in organisms from a wide evolutionary spectrum, including protozoa, yeast, *Drosophila*, fish, and mammals (Figure 1B) [39,42-45]. TbLpn homologues can be identified in other trypanosome genomes such as *Trypanosoma cruzi* and *Leishmania major*, and these proteins display between 32–43.5% amino acid identity with TbLpn [46]. The members of the lipin family serve two major cellular functions: as an enzyme necessary for phospholipid and triacylglycerol biosynthesis, and as a transcriptional cofactor involved in the regulation of lipid metabolism genes [34]. In addition, lipin homologues have been shown to play an essential role in nuclear membrane biogenesis in yeast [47].

**Figure 2:**
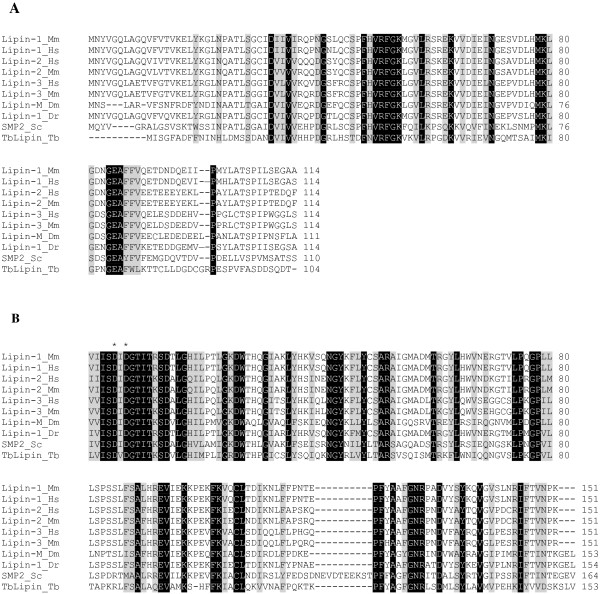
**Amino acid sequence alignment of TbLpn conserved domains and other lipin family members. A**) Amino acid sequence alignment of N-LIP domains. Sequences were aligned using CLUSTALW. Identical and conserved amino acids are shown in black and grey boxes, respectively. **B**) Amino acid sequence alignment of C-LIP domains. Sequences were aligned using CLUSTALW. Identical and conserved amino acids are shown in black and grey boxes, respectively. The conserved aspartic acid residues shown to be essential for enzymatic activity in yeast and mammalian lipins are indicated by asterisks (*).

To begin to characterize TbLpn, we amplified the complete predicted ORF from PF cDNA. Sequence analysis revealed six nucleotide differences from the Tb927.7.5450 sequence reported in GeneDB, three of which result in amino acid changes (Glu-157 → Gly-157, Lys-675 → Thr-675, Val-715 → Ala-715). The predicted TbLpn protein is 806 amino acids in length (Figure 1A) with a predicted molecular mass of 86.7 kDa. The N-LIP domain of TbLpn displays 30–37.5% amino acid identity with the corresponding domains from lipin proteins (Figure 1B and Figure 2A). In addition, the C-LIP domain of TbLpn exhibits 46-50% amino acid identity with the corresponding domains from members of lipin family, such as mammalian lipin-1, lipin-2, lipin-3, and yeast Smp2 (Figure 1B and Figure 2B). Most interesting, the motif (DXDXT) shown to confer phosphatidic acid phosphatase activity to mammalian and yeast lipins, is present within the C-LIP domain of TbLpn (^445^DVDGT) [43]. In addition, a conserved glycine residue shown to be essential for the mouse Lipin-1 function is also present in the predicted amino acid sequence of TbLpn (Gly-74) [39]. Apart from this domain, no significant homology is observed between TbLpn and other members of the lipin family. For instance, although lipin proteins share the LXXIL motif, which has been shown to be essential for interaction of Lipin-1 with the nuclear cofactors involved in the regulation of fatty acid metabolism, TbLpn lacks that conserved LXXIL motif, suggesting that TbLpn might have a different function than other lipins [48]. Although TbLpn may not possess co-transcriptional activity, it might still act as a phosphatidic acid phosphatase. In addition, the conserved nuclear localization sequence, usually found in almost all species [34], is absent in TbLpn.

### Subcellular localization of TbLpn

**Figure 3:**
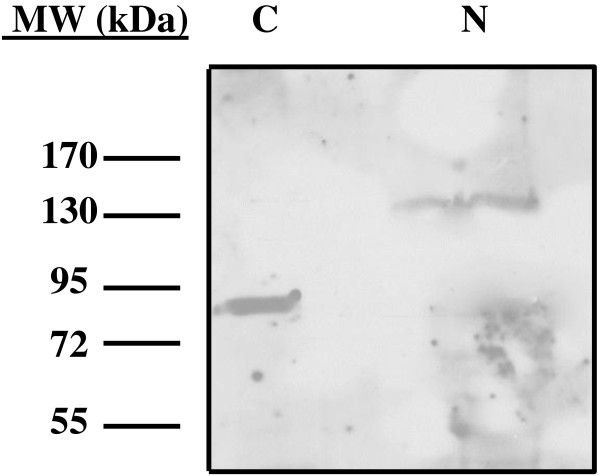
**Analysis of TbLpn subcellular localization.** PF *T. brucei* were fractionated into cytosolic (C) and nuclear (N) extracts as described under Material and Methods. The presence of TbLpn was detected by western hybridization using anti-TbLpn polyclonal antibodies (1:1,000), followed by goat anti-rabbit IgGs, and signals detected using chemiluminescence. Efficiency of the fractionation procedure was assessed by western blot using antibodies against Hsp70 and RNA polymerase II as cytosolic and nuclear markers, respectively.

To determine the subcellular localization of TbLpn, PF *T. brucei* cells were fractionated into cytosolic and nuclear extracts, and the presence of TbLpn within these compartments assessed by western hybridization. The efficiency of the fractionation procedure was confirmed by using antibodies directed against cytosolic Hsp70 and nuclear RNA polymerase II. As shown in Figure 3, a band of the expected size for TbLpn (~ 83 kDa) was present exclusively in the cytoplasm of the parasite. This is in contrast to all previously characterized mammalian and yeast lipins which display cytoplasmic as well as nuclear localization [34,39,49-51]. In addition, SMP2, the yeast lipin homologue, has been shown to be present in the cytosol as well as associated with the membrane [43]. We did however detect the presence of a protein band with decreased electrophoretic mobility (~120 kDa) in the nuclear extract. This strongly suggests that TbLpn is present in both cytosol and nucleus and, in the nucleus, is heavily modified by post-translational modifications such as arginine methylation and/or phosphorylation.

### TbLpn interacts with TbPRMT1 *in vitro* and *in vivo*

**Figure 4:**
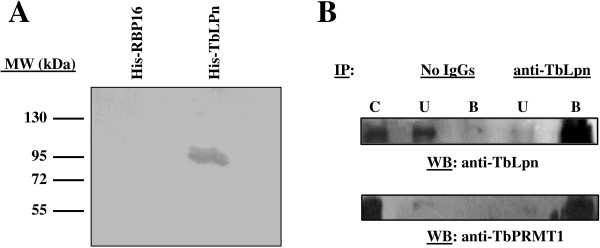
**TbLpn interacts with TbPRMT1. A**) Far western analysis of TbPRMT1-TbLpn interaction. Purified His-TbLpn and His-RBP16 were separated on a 10% polyacrylamide gel, transferred to PVDF, and incubated with purified TbPRMT1 as described under Material and Methods. The blot was probed with anti-TbPRMT1 polyclonal antibodies (1:1,000), followed by goat anti-rabbit IgGs, and signals detected using chemiluminescence. **B**) *In vivo* interaction between TbLpn and TbPRMT1. TbLpn was immunoprecipitated from PF *T. brucei* cytosolic extracts using anti-TbLpn polyclonal antibodies as described under Material and Methods. As a negative control, the cytosolic extract was incubated in the absence of antibodies. Proteins present in the starting cytosolic fraction (C), as well as the bound (B) and unbound fractions (U) were separated on a 10% polyacrylamide gel and transferred to PVDF. The presence of TbLpn in the immune complexes was assessed by probing the membrane with anti-TbLpn polyclonal antibodies (1:1,000), followed by goat anti-rabbit IgGs. The presence of TbPRMT1 in the immune complexes was detected by probing the blot with anti-TbPRMT1 polyclonal antibodies (1:1,000), followed by goat anti-rabbit IgGs. Signals were detected using chemiluminescence.

We further confirmed the TbPRMT1/TbLpn interaction identified by yeast-two-hybrid first by Far Western hybridization. To this end, recombinant His-TbLpn was electrophoresed and transferred to PVDF, and the membrane was incubated with recombinant His-TbPRMT1. Detection of His-TbPRMT1 with polyclonal anti-TbPRMT1 antibodies revealed the presence of a band at 105 kDa, which is the predicted size of His-TbLpn, thereby demonstrating direct binding of His-TbPRMT1 to His-TbLpn (Figure 4A). As a negative control, His-RBP16, expressed and purified using the same protocol as for the purification of His-TbLpn, was used. Using this negative control, no band was detected. The data indicate that TbLpn and TbPRMT1 interact directly.

In order to examine the interaction between TbPRMT1 and TbLpn *in vivo*, we performed a co-immunoprecipitation. As shown above, TbLpn is located in the cytosol of the parasite. For this reason, TbLpn was immunoprecipitated from PF *T. brucei* cytosolic extracts using purified polyclonal anti-TbLpn antibodies. Proteins that were immunoprecipitated along with TbLpn were separated by electrophoresis and transferred onto PVDF. The presence of TPRMT1 in association with TbLpn was determined by using purified polyclonal anti-TbPRMT1 antibodies to probe the membrane by western hybridization. The results shown in Figure 4B clearly show that a band of approximately the size of TbPRMT1 (38.9 kDa) co-precipitates exclusively with TbLpn, and is not present in the negative control.

### TbLpn is methylated *in vivo*

**Figure 5:**
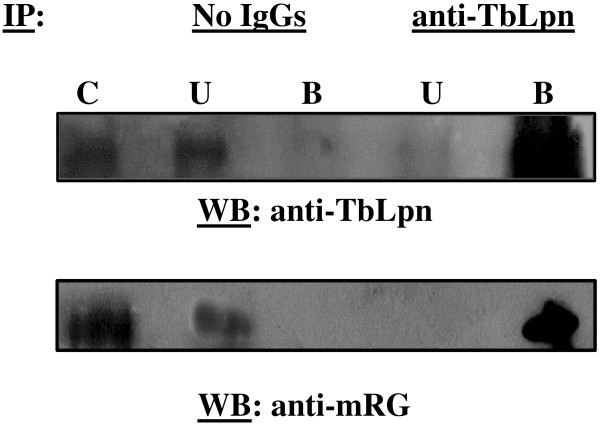
**TbLpn is methylated *in vivo*.** TbLpn was immunopurified from PF *T. brucei* cytosolic extracts using anti-TbLpn polyclonal antibodies as described under Material and Methods. As a negative control, the cytosolic extract was incubated in the absence of antibodies. Proteins present in the starting cytosolic fraction (C), as well as the bound (B) and unbound fractions (U) were separated on a 10% polyacrylamide gel and transferred to PVDF. The presence of TbLpn in the immune complexes was assessed by probing the membrane with anti-TbLpn polyclonal antibodies (1:1,000), followed by goat anti-rabbit IgGs. To determine whether TbLpn contains methylated arginines, the blot was probed with anti-mRG polyclonal antibodies (1:1,000) [52], followed by goat anti-rabbit IgGs. Signals were detected using chemiluminescence.

The physical association of TbPRMT1 with TbLpn suggests that TbLpn may serve as a substrate for methylation by TbPRMT1. In support of this hypothesis, several arginine residues throughout the TbLpn sequence are located within preferred motifs for methylation, such as RG or RXR. To evaluate whether TbLpn is methylated *in vivo*, an immunoprecipitation was performed from PF *T. brucei* cytosolic extracts using purified anti-TbLpn polyclonal antibodies. The presence of methylated arginine residues was then determined by western hybridization using anti-mRG polyclonal antibodies. These antibodies were raised against a peptide containing 7 asymmetric dimethylarginine residues alternating with 8 glycine residues. This motif is found most prevalently among verified dimethylarginine- containing proteins. The antibodies have been shown to specifically recognize methylated arginine residues [52]. Using these antibodies to probe the blot, a protein band was observed at 85 kDa, which is the predicted size of TbLpn, in the bound but not the unbound fraction (Figure 5). This clearly indicates that native TbLpn contains methylated arginine residues. This result, in conjunction with the ability of TbLpn to interact with TbPTMT1 *in vivo*, suggests that one or several arginine residues within TbLpn might be asymmetrically dimethylated by TbPRMT1. Further experiments are underway to identify the enzyme(s) responsible for TbLpn methylation.

### TbLpn displays phosphatidic acid phosphatase activity *in vitro*

**Figure 6:**
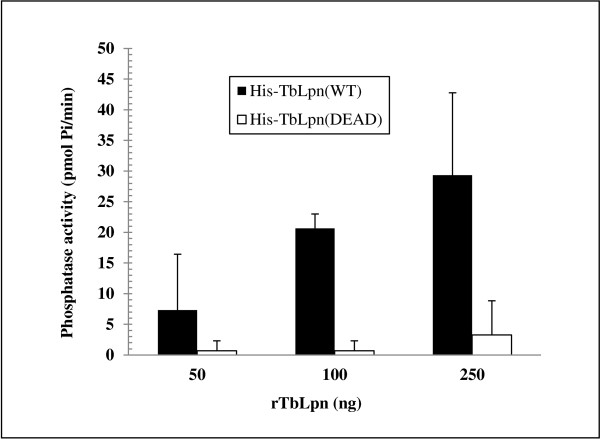
**Recombinant TbLpn displays phosphatidic acid phosphatase activity.** The enzymatic activity was measured by the release of phosphate from 1,2-dioctanoyl-sn-glycero-3-phosphate (DiC8 PA). The substrate was incubated with increasing amounts of either His-TbLpn (black bars) or His-DEAD (white bars) recombinant proteins. The amount of phosphate released was measured using PiBlue reagent and recording the absorbance at 620 nm.

Lipin proteins are known to exhibit Mg^2+^-dependent phosphatidic acid phosphatase activity, catalyzing dephosphorylation of phosphatidic acid (PA) into diacylglycerol. The predicted amino acid sequence of TbLpn contains two conserved domains found in all lipins. In addition, two aspartic acid residues that have been shown to be essential for enzymatic activity of yeast and mammalian lipins are also found in TbLpn. To determine whether recombinant TbLpn could catalyze dephosphorylation of phosphatidic acid, enzymatic assays were performed using the substrate 1,2-dioctanoyl-sn-glycero-3-phosphate (DiC8 PA), Mg^2+^, and increasing amount of His-TbLpn. Following incubation at 30°C, the amount of Pi released was measured by reading the absorbance at 620 nm following the addition of PiBlue reagent. Figure 6 shows that recombinant TbLpn exhibits phosphatidate phosphatase activity, suggesting that TbLpn may play a role in the synthesis of phospholipids. From our data, we calculated that recombinant TbLpn has a specific activity of 200–225 nmol/min/mg. In contrast, the recombinant mutant in which the two conserved aspartic acid residues (Asp-445, Asp-447) were changed to alanines (His-DEAD) shows significantly less phosphatase activity. The calculated specific activity of 11–12 nmol/min/mg calculated for the mutant protein clearly implies that the two conserved aspartates are essential for this enzymatic activity.

## Discussion

In an effort to discover trypanosome substrates of PRMTs, we utilized a yeast two-hybrid screen to identify proteins that associate with the major type I protein arginine methyltransferase in *T. brucei,* TbPRMT1 [27]. Of particular interest to us are proteins whose functions might be affected by arginine methylation. Here, we report that TbPRMT1 directly interacts in both Far Western and co-immunoprecipitation assays with a novel protein. We termed this protein TbLpn, based on the presence of two conserved (N-LIP and C-LIP) domains found in a family of proteins called lipins. We further demonstrate that, like TbPRMT1, TbLpn is cytoplasmic in PF *T. brucei*, consistent with a function in TbLpn methylation. Together, these data point to TbLpn as a candidate protein whose post-transcriptional gene regulatory functions are affected by arginine methylation.

We demonstrated that, as predicted from the amino acid sequence, recombinant TbLpn, as other members of the lipin family, exhibits phosphatidic acid phosphatase enzymatic activity. Mutation of the conserved aspartic acid residues (Asp-445 and Asp- 447) to alanines results in a significant reduction in the enzymatic activity of TbLpn. These two aspartic acid residues are part of the conserved DxDxT motif found in lipin proteins and other members of the haloacid dehalogenase (HAD)-like superfamily [53,54]. Based on the crystal structure of _L_-2-haloacid dehalogenase from *Pseudomonas*, it is likely that Asp-445 in TbLpn acts as a nucleophile in the phosphoryl transfer reaction.

Compared to the recombinant yeast PAH1 (3000 nmol/min/mg) and human Lipin-1 (1,600 nmol/min/mg), His ~ TbLpn displays a lower but still significant specific activity [43]. One possible explanation for this lower specific activity is the fact that the recombinant protein may not contain the same post-translational modifications as those found in the native protein. It is of interest that several lipin homologues are highly modified at the post translational level. In rat and in mouse adipocytes, Lipin 1 contains at least 19 and as many as 23 sites that are phosphorylated in response to insulin [49,55,56]. Although it does not affect its intrinsic phosphatidic acid phosphatase activity, phosphorylation of Lipin-1 decreases the association with intracellular membranes, thus the active lipin fraction [49]. In addition, the lipin homologue SMP2 is phosphorylated by the cyclin-dependent kinase Cdc28/Cdk1 in budding yeast [57]. The authors have shown that phosphorylation of SMP2 by Cdc28/Cdk1 enhances its association with promoters of lipid biosynthetic genes, which leads to their transcriptional down-regulation. Careful analysis of TbLpn amino acid sequence revealed the presence of 5 conserved amino acid residues shown to be phosphorylated in either mouse (Mm) Lipin-1 or yeast (Sc) Smp2. These residues are Ser-102 (Ser-110 in Sc), Thr-239 (Thr-282 in Mm), Thr-255 (Thr-298 in Mm), Ser-282 (Ser-328 in Mm), and Ser-343 (Ser-392 in Mm). In addition, a previous analysis of the cytosolic phosphoproteome of BF *T. brucei* identified TbLpn as containing two phosphorylated residues (Thr-211 and Ser-221) [58]. Whether additional or different amino acids are phosphorylated in the PF is still unclear.

Phosphorylation of TbLpn may also impact its association with other proteins, as it has been demonstrated for at least one other member of the lipin family. In adipocytes, Lipin-1 interacts directly with 14-3-3 proteins [51]. 14-3-3- proteins are known to regulate the subcellular localization of a wide variety of proteins through interaction with phosphoserine residues. In adipocytes, Lipin-1 is mostly localized to the cytosol and translocate to the endoplasmic reticulum membrane where it catalyzes dephosphorylation of phosphatidic acid. Stimulation of adipocytes by insulin promotes phosphorylation of Lipin-1 and enhances binding by 14-3-3 proteins. This results in cytoplasmic retention of Lipin-1.

Additionally, we showed that TbLpn is methylated on arginine residues *in vivo*. To our knowledge, this is the first instance of any lipin or phosphatidic acid phosphatase being methylated. The demonstration that TbLpn is methylated *in vivo* suggests that methylation could directly modulate TbLpn enzymatic activity or protein-protein interactions, or both. Arginine methylation has been shown to generally alter protein function by modulating protein-protein interactions, protein-nucleic acid interactions, and protein trafficking [11,21,59,60]. Arginine residues that serve as substrates for PRMTs are usually found within glycine/arginine rich (GAR) domains [61-63]. Based on this, arginine residues throughout TbLpn, including both the N-LIP and C-LIP domains are predicted to undergo methylation. Thus, it will be of great future interest to determine whether TbPRMT1 and/or other TbPRMTs are responsible for TbLpn methylation *in vivo*, and to determine whether TbLpn methylation has any effect on its ability to interact with other proteins and whether it modulates its enzymatic activity.

In yeast and mammals, lipin proteins enable the cell to generate diacylglycerol (DAG) by catalyzing the dephosphorylation of phosphatidic acid. In addition to serving as a precursor for triacylglycerol (TAG), DAG is also used to synthesize phosphatidylcholine (PC) and phosphatidylethanolamine (PE) [64]. In mammalian and yeast cells, the bulk of the PC pool is synthesized by the CDP-choline branch of the Kennedy pathway [64]. In addition, a small fraction of PC is generated by sequential methylation of PE [64]. In eukaryotes, PE can be synthesized by decarboxylation of phosphatidylserine (PS), by head group exchange with PS, by acylation of lyso-PE, or by the CDP-ethanolamine branch of the Kennedy pathway [65,66]. As for other eukaryotes, PC and PE constitute the majority of phospholipids in trypanosomes [67]. Of great importance is the fact that, as opposed to other parasitic organisms, trypanosomes synthesize phospholipids *de novo*[68]. Although the pathways for phospholipids biosynthesis have not been very well characterized, recent data have helped to better understand how trypanosomes are able to assemble phospholipids. *In T. brucei*, PC is synthesized solely by the CDP-choline branch of the Kennedy pathway, while PE is produced exclusively via the CDP-ethanolamine branch of the Kennedy pathway [67,69,70]. Disruption of the enzymes of the CDP-ethanolamine pathway by RNA interference have shown that this branch of the Kennedy pathway is essential for both procyclic and bloodstream form *T. brucei* cell growth [69,71].

PE and phosphatidylinositol (PI) are key phospholipids involved in the biosynthesis of glycosylphosphatidylinositol (GPI). In trypanosomes, a large number of surface proteins with critical role in virulence surface proteins are anchored to the plasma membrane via GPI molecules. One of these proteins is the variant surface glycoprotein (VSG), a major virulence factor that undergoes antigenic variation and enables the parasite to evade the immune system of its mammalian host [70]. The steps involved in the biosynthesis of GPI, a process essential for *T. brucei* bloodstream form survival, have been well studied. This synthesis differs in certain aspects from the pathway in mammalian cells and yeast. In *T. brucei*, the pool of PI used for GPI synthesis is supplied from glucose-6-phosphate by the action of PI synthase, an enzyme shown to be essential in both bloodstream and procyclic form trypanosomes [68,70,71]. A crucial step in the GPI synthesis pathway is the transfer of phosphoethanolamine (PEtN) to mannose residues on the growing GPI. In this reaction, the ethanolamine moiety is provided by PE [72]. As described earlier, synthesis of PE in *T. brucei* is carried out via the CDP-ethanolamine branch of the Kennedy pathway using DAG as the initial substrate. It has been demonstrated that inhibition of PE synthesis prevents *de novo* GPI biosynthesis [73]. As we demonstrated in the current paper that TbLpn catalyzes the dephosphorylation of PA into DAG, it is attractive to speculate that TbLpn plays an important role in GPI biosynthesis, and thus in the expression of this major virulence factor.

## Conclusion

The results clearly identify TbLpn as a new member of the lipin family of proteins. The presence of the conserved N-LIP and C-LIP domains, and especially the ability of recombinant TbLpn to dephosphorylate phosphatidic acid indicate that this enzyme is likely to be involved in phospholipid biosynthesis in trypanosomes. Finally, the observation that, *in vivo*, TbLpn contains methylated arginine residues is very significant, as it is the only lipin or phosphatidic acid phosphatase to date to exhibit such a post-translational modification.

## Methods

### Trypanosome growth

Procyclic form *T. brucei brucei* clone IsTaR1 stock EATRO 164 was grown as described in SDM-79 medium supplemented with 15% fetal bovine serum (FBS) [74].

### Identification of TbLpn by yeast two-hybrid screening

For two-hybrid screening, the TbPRMT1 open reading frame (ORF) was amplified by 35 cycles of PCR from pMal-TbPRMT1 [27] using primers PRMT1-Y2H/myc-5′ (5′-GCTCTAGACATATGACGGTGGACGCAAATGCCG-3′) and PRMT-Y2H/myc-3′ (5′-GCGGATCCCTATCTAGACCGCAGCCGAAAATCCTGGTC-3′) which allowed introduction of *Nde*I and *Bam*HI restriction sites respectively (underlined). The PCR product was then cloned into *Nde*I and *Bam*HI sites of pAS2-1 (CLONTECH Laboratories), and transformed into *Escherichia coli* DH5α competent cells (Invitrogen).

The bait plasmid pAS2-TbPRMT1 was co-transformed into the competent yeast strain AH109, along with a mixed procyclic and bloodstream form *T. brucei* cDNA library (a generous gift from George Cross, Rockefeller Univ. and Vivian Bellofatto, UMDNJ) cloned into pGADT7 (CLONTECH Laboratories) using the LiAc/PEG method [75]. Transformed cells were plated onto synthetic dextrose medium (SD) supplemented with an amino acid dropout solution lacking histidine (His), leucine (Leu), and tryptophan (Trp) and incubated at 30°C. Resultant colonies were then streaked onto SD medium lacking His, Leu, Trp, and adenine (Ade). Colonies that grew on this medium were grown overnight at 30°C in 3 ml of SD broth lacking Leu. Cells were collected by centrifugation at 14,000 × rpm for 5 min in a Biofuge centrifuge. The pellet was resuspended in about 50 μl of residual liquid, and 10 μl of a 10 units/μl lyticase solution was added and thoroughly mixed. Cell lysis was allowed to proceed at 37°C for 60 min with shaking at 250 rpm. Twenty μl of 10% SDS was then added and the tube vortexed for 1 min. The samples were then put to a freeze/thaw cycle (at -20°C) and vortexed one more time. The plasmid was purified using a GFX DNA purification column (GE Healthcare) following the manufacturer’s instructions, and eluted with 50 μl of deionized water. Five μl of the purified plasmid was used to transform 20 μl of ELECTROMAX DH10B cells (Invitrogen). Briefly, electroporation was carried out on ice in 2-mm cuvettes using a Bio-Rad electroporator with the following settings: 2,000 V, 25 μF, 200 Ω. Following electroporation, 1 ml of SOC was added and the cells were transferred to a 15-ml snap cap tube, and incubated for 60 min at 37°C with shaking (250 rpm). Fifty and 500 μl were then plated onto LB plates containing 0.1 mg/ml ampicillin, and cells were allowed to grow at least 18 hours at 37°C. Colonies with pGADT7 containing a DNA fragment were identified by PCR using primers GAL4AD5′ (5′-CAGGGATGTTTAATACCACTA-3′) and GAL4AD3′ (5′-GCACAGTTGAAGTGAACTTGC-3′), and sequenced.

### Production of recombinant TbLpn

C-terminally his-tagged TbLpn was generated as follows. Total PF cDNA was generated by reverse transcription primed with [dT]-RXS. The entire TbLpn ORF was amplified using Deep Vent DNA polymerase (New England Biolabs), and using oligonucleotides his10-lipin-5′ (5′-CGGGATCCATGATATCTGGTTTTGCAGATTTC-3′) and his10-lipin3′ (5′-CCCAAGCTTCCGCTCGAGTCACACAGTGTCACCTTGTTGATA-3′) (restriction sites are underlined) which were constructed based on the genomic sequence. The PCR product was then digested with *Bam*HI and *Xho*I, ligated into the pET26-His_10_Smt3 expression vector, giving rise to pHis10-TbLpn, and transformed into *E. coli* BL21 competent cells (Invitrogen). A mutant version of TbLpn, in which the two conserved aspartic acid residues in the DVDGT motif (Asp-445, Asp-447) are changed to alanine (pHis-TbLpn(DEAD)), was generated by PCR amplification from pHis10-TbLpn using the QuikChange II XL™ Site-Directed Mutagenesis Kit (Agilent Technologies) and the mutagenic primers TbLpn-DEAD-5′ (5′-CTTGTCATTAGTGAAGTGGAAGGCACGATCACGAAAAG-3′) and TbLpn-DEAD-3′ (5′-CTTTTCGTGATCGTGCCTTCCACTTCACTAATGACAAG-3′).

Protein expression was induced with 1 mM isopropyl β-thiogalactopyranoside (IPTG) and 2% ethanol for 20 h at 17°C. Cells were resuspended in lysis buffer (10 mM Tris [pH 8.6], 10 mM glycine, 300 mM NaCl, 10 mM imidazole, 10% glycerol, 10% ethanol, 4% Tween-20, and 3% Triton X-100) containing 0.05 mg/ml lysozyme, 0.01 mg/ml DNase I, 1 mM phenylmethylsulfonyl fluoride (PMSF), 1 μg/ml leupeptin, and 1μg/ml pestatin A, and lysed by 3 freeze/thaw cycles. Each cycle consisted of incubation at 37°C for 15 minutes, followed by incubation at -80°C for another 15 minutes. The lysed cell suspension was centrifuged at 17,000 × g for 15 min at 4°C, and the supernatant was mixed with Probond Ni^2+^ resin (Invitrogen) for 12 h at 4°C. The mixture was poured into a column and the column washed with 40 volumes of wash buffer (10 mM Tris [pH 7.0], 200 mM NaCl, 30 mM imidazole, 10% glycerol). His-tagged proteins were eluted with 10 volumes of wash buffer (pH 6.0) containing 200 mM imidazole.

### Polyclonal antibody production

Affinity purified polyclonal anti-TbLpn antibodies were obtained from Bethyl Laboratories, Inc. using a peptide corresponding to amino acids 791–806 (GLCNTSSENYQQGDTV).

### Far western analysis

His-tagged TbLpn was electrophoresed on a denaturing 10% SDS-polyacrylamide gel and transferred onto a polyvinylidene fluoride (PVDF) membrane at 50 V for 45 min in 10 mM 3-[Cyclohexylamino]-1-propanesulfonic acid (CAPS) buffer (pH 11.0) containing 10% methanol. As a negative control, his-tagged RBP16 was expressed as described [76] and purified using the same protocol used for the purification of His-TbLpn described above. The membrane was blocked in TBS buffer containing 5% nonfat dry milk for 1 hour, washed twice for 5 min in TBS buffer containing 0.05% Tween-20 (TBS-T), and then incubated with 0.5-1.0 μg of purified TbPRMT1 [27] in TBS-T containing 2% nonfat dry milk overnight at 4°C. After two 15 minute washes in TBS-T, the membrane was probed with anti-TBPRMT1 polyclonal antibodies (1:1,000) for 2 hours, washed in TBS-T twice for 15 min, and incubated with goat anti-rabbit IgGs coupled to horseradish peroxidase. Reactive proteins were detected using enhanced chemiluminescence (GE Healthcare).

### Preparation and fractionation of trypanosome cellular extracts

Log-phase PF *T. brucei brucei* clone IsTaR1 stock EATRO 164 were harvested by centrifugation at 6,090 × g for 10 min at 4°C. Fractionation of trypanosome cellular extracts was performed as described previously [77]. The integrity of the cellular compartment was confirmed by using antibodies directed against the cytosolic protein Hsp70 or the nuclear RNA polymerase II [78].

### Immunoprecipitation of TbLpn from *T. brucei* cytosolic extracts

As it was previously determined that TbLpn is localized in the cytosol, immunoprecipitation of TbLpn was performed using PF form *T. brucei* cytosolic extracts. Ten μg of purified anti-TbLpn antibodies or 10 μl of IP buffer (for mock immunoprecipitations) (20 mM Hepes [pH 7.9], 150 mM sucrose, 150 mM KCl, 3 mM MgCl_2_, 0.5% Nonidet- P40, 1 μg/ml of pestatin A, 1 μg/ml of leupeptin, 5 mM PMSF) were added to 200 μl of cytosolic extract in a final volume of 300 μl of IP buffer. The samples were incubated at 4°C for at least 12 h with gentle rotation. Ten μl of Protein A-Sepharose (GE Healthcare) was then added, and the samples incubated 1 hour at 4°C with gentle rotation. Immune complexes were recovered by centrifugation at 3,000 × *g* for 30 s and washed five times, each time for 5 min, with 1 ml of IP buffer.

### Phosphatidic acid phosphatase assays

The standard reaction contained 50 mM Tris–HCl buffer (pH 7.5), 1 mM MgCl_2_, and 0.4 mM 1,2-dioctanoyl-*sn*-glycero-3-phosphate (DiC8 PA) (Avanti Polar Lipids) in a total volume of 50 μl. Reactions were initiated by the addition of recombinant proteins (50–250 ng), and carried out in triplicate at 30°C for 30 min. The reaction was terminated by the addition of 100 μl of PiBlue reagent (BioAssay Systems), and the color allowed to develop at room temperature for 30 minute. The absorbance was measured with a spectrophotometer at 620 nm. The amount of phosphate produced was quantified from a standard curve using 0.5–4 nmol of potassium phosphate. The reactions were linear with time and protein concentration. The enzymatic activity was expressed as the number of pmol of phosphate released per minute.

## Competing interest

The authors declare that they have no competing interest.

## Authors’ contribution

MP designed the study. MP performed the yeast-two hybrid screening and analysis. JMW performed the subcellular fractionation and localization assays. JSS and DNM expressed and purified wild type His ~ TbLpn. ARK performed the site-directed mutagenesis, expressed, and purified the His ~ DEAD mutant. ASF contributed by performing immunoprecipitation and western hybridization analyses. The *in vitro* phosphatidic acid phosphatase assays were performed by MP, DNM, and ARK. MP wrote the manuscript. All authors read and approved the final manuscript.

## References

[B1] Bachand F Protein arginine methyltransferases: from unicellular eukaryotes to humans Eukaryot Cell 2007 6 889 898 10.1128/EC.00099-07 17468392 PMC1951521

[B2] Bedford MT Arginine methylation at a glance J Cell Sci 2007 120 4243 4246 10.1242/jcs.019885 18057026

[B3] Bedford MT Clarke SG Protein arginine methylation in mammals: who, what, and why Mol Cell 2009 33 1 13 10.1016/j.molcel.2008.12.013 19150423 PMC3372459

[B4] Krause CD Yang ZH Kim YS Lee JH Cook JR Pestka S Protein arginine methyltransferases: evolution and assessment of their pharmacological and therapeutic potential Pharmacol Ther 2007 113 50 87 10.1016/j.pharmthera.2006.06.007 17005254

[B5] Boisvert FM Chénard CA Richard S Protein interfaces in signaling regulated by arginine methylation Sci Signal 2005 271 re2 10.1126/stke.2712005re2 15713950

[B6] Weber S Maaβ F Schuemann M Krause E Suske G Bauer UM PRMT1-mediated arginine methylation of PIAS1 regulated STAT1 signaling Genes Dev 2009 23 118 132 10.1101/gad.489409 19136629 PMC2632166

[B7] Green DM Marfatia KA Crafton EB Zhang X Cheng X Corbett AH Nab2p is required for poly(A) RNA export in *Saccharomyces cerevisiae* and is regulated by arginine methylation via Hmt1p J Biol Chem 2002 277 7752 7760 10.1074/jbc.M110053200 11779864

[B8] Lukong KE Richard S Arginine methylation signals mRNA export Nat Struct Mol Biol 2004 11 914 915 10.1038/nsmb1004-914 15452560

[B9] Godin KS Varani G How arginine-rich domains coordinate mRNA maturation events RNA Biol 2007 4 69 75 10.4161/rna.4.2.4869 17873524

[B10] Polevoda B Sherman F Methylation of proteins involved in translation Mol Micro 2007 65 590 606 10.1111/j.1365-2958.2007.05831.x 17610498

[B11] Yu MC Bachand F McBride AE Komili S Casolari JM Silver PA Arginine methyltransferase affects interactions and recruitment of mRNA processing and export factors Genes Dev 2004 18 2024 2035 10.1101/gad.1223204 15314027 PMC514182

[B12] Xie B Invernizzi CF Richard S Wainberg MA Arginine methylation of the human immunodeficiency virus type 1 Tat protein by PRMT6 negatively affects Tat interactions with both cyclin T1 and the Tat transactivation region J Virol 2007 81 4226 4234 10.1128/JVI.01888-06 17267505 PMC1866113

[B13] De Leeuw F Zhang T Wauquier C Huez G Kruys V Gueydan C The cold-inducible RNA-binding protein migrates from the nucleus to cytoplasmic stress granules by a methylation-dependent mechanism and acts as a translational repressor Exp Cell Res 2007 313 4130 4144 10.1016/j.yexcr.2007.09.017 17967451

[B14] Perreault A Lemieux C Bachand F Regulation of the nuclear poly(A)-binding protein by arginine methylation in fission yeast J Biol Chem 2007 282 7552 7562 17213188 10.1074/jbc.M610512200

[B15] Smith WA Schurter BT Wong-Staal F David M Arginine methylation of RNA helicase A determines its subcellular localization J Biol Chem 2004 279 22795 22798 10.1074/jbc.C300512200 15084609

[B16] Lee DY Teyssier C Strahl BD Stallcup MR Role of protein methylation in regulation of transcription Endocr Rev 2005 26 147 170 10.1385/ENDO:26:2:147 15479858

[B17] Côté J Boisvert FM Boulanger MC Bedford MT Richard S Sam68 RNA Binding Protein Is an In Vivo Substrate for Protein Arginine N-Methyltransferase 1 Mol Biol Cell 2003 14 274 287 10.1091/mbc.E02-08-0484 12529443 PMC140244

[B18] Goulah CC Read LK Differential effects of arginine methylation on RBP16 mRNA binding, guide RNA (gRNA) binding, and gRNA-containing ribonucleoprotein complex (gRNP) formation J Biol Chem 2007 282 7181 7190 17229732 10.1074/jbc.M609485200

[B19] McBride AE Cook JT Stemmler EA Rutledge KL McGrath KA Rubens JA Arginine methylation of yeast mRNA-binding protein Npl3 directly affects its function, nuclear export, and intranuclear protein interactions J Biol Chem 2005 280 30888 30898 10.1074/jbc.M505831200 15998636

[B20] Stetler A Winograd C Sayegh J Cheever A Patton E Zhang X Clarke S Ceman S Identification and characterization of the methyl arginines in the fragile X mental retardation protein Fmrp Hum Mol Genet 2005 15 87 96 10.1093/hmg/ddi429 16319129

[B21] Bedford MT Richard S Arginine methylation: An emerging regulator of protein function Mol Cell 2005 18 263 272 10.1016/j.molcel.2005.04.003 15866169

[B22] McBride AE Silver PA State of the Arg: Protein methylation at arginine comes of age Cell 2001 106 5 8 10.1016/S0092-8674(01)00423-8 11461695

[B23] Pahlich S Zakaryan RP Gehring H Protein arginine methylation: Cellular functions and methods of analysis Biochim Biophys Acta 2006 1764 1890 1903 10.1016/j.bbapap.2006.08.008 17010682

[B24] Wooderchak WL Zang T Zhou ZS Acuña M Tahara SM Hevel JM Substrate profiling of PRMT1 reveals amino acid sequences that extend beyond the “RGG” paradigm Biochemistry 2008 47 9456 9466 10.1021/bi800984s 18700728

[B25] Wolf SS The protein arginine methyltransferase family: an update about function, new perspectives and the physiological role in humans Cell Mol Life Sci 2009 66 2109 2121 10.1007/s00018-009-0010-x 19300908 PMC11115746

[B26] Fisk JC Read LK Protein arginine methylation in parasitic protozoa Eukaryot Cell 2011 10 1013 1022 10.1128/EC.05103-11 21685318 PMC3165437

[B27] Pelletier M Pasternack DA Read LK *In vitro* and *in vivo* analysis of the major type I protein arginine methyltransferase from *Trypanosoma brucei* Mol Biochem Parasitol 2005 144 206 217 10.1016/j.molbiopara.2005.08.015 16198009

[B28] Pasternack DA Sayegh J Clarke S Read LK Evolutionarily divergent type II protein arginine methyltransferase in *Trypanosoma brucei* Eukaryot Cell 2007 6 1665 1681 10.1128/EC.00133-07 17601874 PMC2043365

[B29] Fisk JC Sayegh J Zurita-Lopez C Menon S Presnyak V Clarke SG Read LK A type III protein arginine methyltransferase from the protozoan parasite *Trypanosoma brucei* J Biol Chem 2009 284 11590 11600 19254949 10.1074/jbc.M807279200 PMC2670164

[B30] Fisk JC Zurita-Lopez C Sayegh J Tomasello DL Clarke SG Read LK TbPRMT6 is a type I protein arginine methyltransferase that contributes to cytokinesis in *Trypanosoma brucei* Eukaryot Cell 2010 9 866 877 10.1128/EC.00018-10 20418380 PMC2901642

[B31] Goulah CC Pelletier M Read LK Arginine methylation regulates mitochondrial gene expression in Trypanosoma brucei through multiple effector proteins RNA 2006 12 1545 1555 10.1261/rna.90106 16775306 PMC1524885

[B32] Berriman M Ghedin E Hertz-Fowler C Blandin G Renauld H Bartholomeu DC Lennard NJ Caler E Hamlin NE Haas B Böhme U Hannick L Aslett MA Shallom J Marcello L Hou L Wickstead B Alsmark UC Arrowsmith C Atkin RJ Barron AJ Bringaud F Brooks K Carrington M Cherevach I Chillingworth TJ Churcher C Clark LN Corton CH Cronin A The genome of African trypanosome *Trypanosoma brucei* Science 2005 309 416 422 10.1126/science.1112642 16020726

[B33] Passos DO Bressan GC Nery FC Kobarg J Ki-1/57 interacts with PRMT1 and is a substrate for arginine methylation FEBS J 2006 273 3946 3961 10.1111/j.1742-4658.2006.05399.x 16879614

[B34] Reue K Zhang P The lipin protein family: dual roles in lipid biosynthesis and gene expression FEBS Lett 2008 582 90 96 10.1016/j.febslet.2007.11.014 18023282 PMC2848953

[B35] Harris TE Finck BN Dual function lipin proteins and glycerolipid metabolism Trends Endocrinol Metab 2011 22 226 233 10.1016/j.tem.2011.02.006 21470873 PMC3118913

[B36] Inoue K Mizuno T Wada K Hagiwara M Novel RING Finger proteins, Air1p and Air2p, interact with Hmt1p and inhibit the arginine methylation of Npl3p J Biol Chem 2000 275 32793 32799 10896665 10.1074/jbc.M004560200

[B37] Tang J Kao PN Herschman HR Protein-arginine methyltransferase I, the predominant protein-arginine methyltransferase in cells, interacts with and is regulated by interleukin enhancer-binding factor 3 J Biol Chem 2000 275 19866 19876 10.1074/jbc.M000023200 10749851

[B38] Hoek M Zanders T Cross GAM *Trypanosoma brucei* expression-site-associated-gene-8 protein interacts with a Pumilio family protein Mol Biochem Parasitol 2002 120 269 283 10.1016/S0166-6851(02)00009-9 11897132

[B39] Péterfy M Xu P Reue K Phan Lipodystrophy in the fld mouse results from mutation of a new gene encoding a nuclear protein, lipin Nat Genet 2001 27 121 124 10.1038/83685 11138012

[B40] Langner CA Birkenmeier EH Roth KA Bronson RT Gordon JI Characterization of the peripheral neuropathy in neonatal and adult mice that are homozygous for the fatty liver dystrophy (*fld*) mutation J Biol Chem 1991 266 11955 11964 2050689

[B41] Reue K Xu P Wang XP Slavin BG Adipose tissue deficiency, glucose intolerance, and increased atherosclerosis result from mutation in the mouse fatty liver dystrophy (*fld*) gene J Lipid Res 2000 41 1067 1076 10884287

[B42] Donkor J Sariahmetoglu M Dewald J Brindley DN Reue K Three mammalian lipins act as phosphatidate phosphatases with distinct tissue expression patterns J Biol Chem 2007 282 3450 3457 17158099 10.1074/jbc.M610745200

[B43] Han GS Wu WI Carman GM The *Saccharomyces cerevisiae* Lipin homolog is a Mg2 + -dependent phosphatidate phosphatase enzyme J Biol Chem 2006 281 9210 9218 16467296 10.1074/jbc.M600425200 PMC1424669

[B44] Rupali U Liu Y Provaznik J Schmitt S Lehmann M Lipin Is a Central Regulator of Adipose Tissue Development and Function in *Drosophila melanogaster* Mol Cell Biol 2011 31 1646 1656 10.1128/MCB.01335-10 21300783 PMC3126333

[B45] Strausberg RL Feingold EA Grouse LH Derge JG Klausner RD Collins FS Wagner L Shenmen CM Schuler GD Altschul SF Zeeberg B Buetow KH Schaefer CF Bhat NK Hopkins RF Jordan H Moore T Max SI Wang J Hsieh F Diatchenko L Marusina K Farmer AA Rubin GM Hong L Stapleton M Soares MB Bonaldo MF Casavant TL Scheetz TE Generation and initial analysis of more than 15,000 full-length human and mouse cDNA sequences Proc Natl Acad Sci USA 2002 99 16899 16903 10.1073/pnas.242603899 12477932 PMC139241

[B46] El-Sayed NM Myler PJ Bartholomeu DC Nilsson D Aggarwal G Tran AN Ghedin E Wourthey EA Delcher AL Blandin G Westenberger SJ Caler E Cerqueira GC Branche C Haas B Anupama A Arner E Aslund L Attipoe P Bontempi E Bringaud F Burton P Cadag E Campbell DA Carrington M Crabtree J Darban H da Silveira JF de Jong P Edwards K The genome sequence of Trypanosoma cruzi, etiologic agent of Chagas disease Science 2005 309 409 415 10.1126/science.1112631 16020725

[B47] Siniossoglou S Lipins, lipids and nuclear envelope structure Traffic 2009 10 1181 1187 10.1111/j.1600-0854.2009.00923.x 19490535

[B48] Finck BN Gropler MC Chen Z Leone TC Croce MA Harris TE Lawrence JC Jr Kelly DP Lipin 1 is an inducible amplifier of the hepatic PGC-1alpha/PPARalpha regulatory pathway Cell Metab 2006 4 199 210 10.1016/j.cmet.2006.08.005 16950137

[B49] Harris TE Huffman TA Chi A Shabanowitz J Hunt DF Kumar A Lawrence JC Jr Insulin controls subcellular localization and multisite phosphorylation of the phosphatidic acid phosphatase, lipin 1 J Biol Chem 2007 282 277 286 17105729 10.1074/jbc.M609537200

[B50] Péterfy M Phan J Reue K Alternatively spliced lipin isoforms exhibit distinct expression pattern, subcellular localization, and role in adipogenesis J Biol Chem 2005 280 32883 32889 10.1074/jbc.M503885200 16049017

[B51] Péterfy M Harris TE Fujita N Reue K Insulin-stimulated interaction with 14-3-3 promotes cytoplasmic localization of lipin-1 in adipocytes J Biol Chem 2010 285 3857 3864 10.1074/jbc.M109.072488 19955570 PMC2823528

[B52] Duan P Xu Y Birkaya B Myers J Pelletier M Read LK Guarnaccia C Pongor S Denman RB Aletta JM Generation of polyclonal antiserum for the detection of methylarginine proteins J Immunol Methods 2007 320 132 142 10.1016/j.jim.2007.01.006 17307197 PMC1950451

[B53] Koonin EV Tatusov RL Computer analysis of bacterial haloacid dehalogenases defines a large superfamily of hydrolases with diverse specificity. Application of an iterative approach to database search J Mol Biol 1994 244 125 132 10.1006/jmbi.1994.1711 7966317

[B54] Hisano T Hata Y Fujii T Liu JQ Kurihara T Esaki N Soda K Crystal structure of L-2 haloacid dehalogenase from Pseudomonas sp. YL J Biol Chem 1996 34 20322 20330 8702766 10.1074/jbc.271.34.20322

[B55] Huffman TA Mothe-Satney I Lawrence JC Jr Insulin-stimulated phosphorylation of lipin mediated by the mammalian target of rapamycin Proc Natl Acad Sci USA 2002 99 1047 1052 10.1073/pnas.022634399 11792863 PMC117427

[B56] O’Hara L Han G-S Peak-Chew S Grimsey N Carman GM Siniossoglou S Control of phospholipid synthesis by phosphorylation of the yeast lipin Pah1p/Smp2p Mg^2+^-dependent phosphatidate phosphatase J Biol Chem 2006 281 34537 34548 10.1074/jbc.M606654200 16968695 PMC1769310

[B57] Santos-Rosa H Leung J Grimsey N Peak-Chew S Siniossoglou S The yeast lipin Smp2 couples phospholipid biosynthesis to nuclear membrane growth EMBO J 2005 24 1931 1941 10.1038/sj.emboj.7600672 15889145 PMC1142606

[B58] Nett IRE Martin DMA Miranda-Saavedra D Lamont D Barber JD Mehlert A Ferguson MAJ The phosphoproteome of bloodstream form *Trypanonosoma brucei*, causative agent of African Sleeping Sickness Mol Cell Proteomics 2009 8 1527 1538 10.1074/mcp.M800556-MCP200 19346560 PMC2716717

[B59] Cheng D Côté J Shaaban S Bedford MT The arginine methyltransferase CARM1 regulates the coupling of transcription and mRNA processing Mol Cell 2007 25 71 83 10.1016/j.molcel.2006.11.019 17218272

[B60] Côté J Richard S Tudor domains bind symmetrical dimethylated arginines J Biol Chem 2005 280 28476 28483 10.1074/jbc.M414328200 15955813

[B61] Kim S Merrill BM Rajpurohit R Kumar A Stone KL Papov VV Schneiders JM Szer W Wilson SH Paik WK Williams KR Identification of N(G)-methylarginine residues in human heterogeneous RNP protein A1: Phe/Gly-Gly-Gly-Arg-Gly-Gly-Gly/Phe is a preferred recognition motif Biochemistry 1997 36 5185 5192 10.1021/bi9625509 9136880

[B62] Liu Q Dreyfuss G *In vivo* and *in vitro* arginine methylation of RNA-binding proteins Mol Cell Biol 1995 15 2800 2808 7739561 10.1128/mcb.15.5.2800 PMC230511

[B63] Najbauer J Johnson BA Young AL Aswad DW Peptides with sequences similar to glycine, arginine-rich motifs in proteins interacting with RNA are efficiently recognized by methyltransferase(s) modifying arginine in numerous proteins J Biol Chem 1993 268 10501 10509 7683681

[B64] Vance JE Vance DE Phospholipid biosynthesis in mammalian cells Biochem Cell Biol 2004 82 113 128 10.1139/o03-073 15052332

[B65] Kennedy EP Weiss SB The function of cytidine coenzymes in the biosynthesis of phospholipids J Biol Chem 1956 222 193 214 13366993

[B66] Vance JE Steenbergen R Metabolism and functions of phosphatidylserine Prog Lipid Res 2005 44 207 234 10.1016/j.plipres.2005.05.001 15979148

[B67] Smith TK Bütikofer P Lipid metabolism in *Trypanosoma brucei* Mol Biochem Parasitol 2010 172 66 79 10.1016/j.molbiopara.2010.04.001 20382188 PMC3744938

[B68] Martin KL Smith TK Phosphatidylinositol synthesis is essential in bloodstream form *Trypanosoma brucei* Biochem J 2006 396 287 295 10.1042/BJ20051825 16475982 PMC1462709

[B69] Signorell A Rauch M Jelk J Ferguson MAJ Bütikofer P Phosphatidylethanolamine in *Trypanosoma brucei* is organized in two separate pools and is synthesized exclusively by the Kennedy Pathway J Biol Chem 2008 283 23636 23644 10.1074/jbc.M803600200 18587155 PMC3259767

[B70] Ferguson MAJ The structure, biosynthesis and functions of glycosylphosphatidylinositol anchors, and the contributions of trypanosome research J Cell Science 1999 112 2799 2809 10444375 10.1242/jcs.112.17.2799

[B71] Martin KL Smith TK The glycosylphosphatidylinositol (GPI) biosynthetic pathway of bloodstream form *Trypanosoma brucei* is dependent on the *de novo* synthesis of inositol Mol Microbiol 2006 61 89 105 10.1111/j.1365-2958.2006.05216.x 16824097 PMC3793301

[B72] Menon AK Eppinger M Mayor S Schwarz RT Phosphatidylethanolamine is the donor of the terminal phosphoethanolamine group in trypanosome glycosylphosphatidylinositols EMBO J 1993 12 1907 1914 8491183 10.1002/j.1460-2075.1993.tb05839.x PMC413411

[B73] Gibellini F Hunter WN Smith TK The ethanolamine branch of the Kennedy pathway is essential in the bloodstream form of *Trypanosoma brucei* Mol Microbiol 2009 73 826 843 10.1111/j.1365-2958.2009.06764.x 19555461 PMC2784872

[B74] Brun R Schonenberg M Cultivation and *in vitro* cloning of procyclic culture forms of *Trypanosoma brucei* in a semi-defined medium Acta Trop 1979 36 289 292 43092

[B75] Gietz D St-Jean A Woods RA Schiestl RH Improved method for high efficiency transformation of intact yeast cells Nucleic Acids Res 1992 20 1425 10.1093/nar/20.6.1425 1561104 PMC312198

[B76] Hayman ML Miller MM Chandler DM Goulah CC Read LK The trypanosome homolog of human p32 interacts with RBP16 and stimulates its gRNA binding activity Nucleic Acids Res 2001 29 5216 5225 10.1093/nar/29.24.5216 11812855 PMC97595

[B77] Zeiner GM Sturm NR Campbell DA Exportin 1 mediates nuclear export of the kinetoplastid spliced leader RNA Eukaryot Cell 2003 2 222 230 10.1128/EC.2.2.222-230.2003 12684371 PMC154853

[B78] Chapman AB Agabian N *Trypanosoma brucei* RNA polymerase II is phosphorylated in the absence of carboxyl-terminal domain heptapeptide repeats J Biol Chem 1994 269 7 4754 4760 8106443

